# A Practical Guide to the Automated Analysis of Vascular Growth, Maturation and Injury in the Brain

**DOI:** 10.3389/fnins.2020.00244

**Published:** 2020-03-20

**Authors:** Ruslan Rust, Tunahan Kirabali, Lisa Grönnert, Berre Dogancay, Yanuar D. P. Limasale, Andrea Meinhardt, Carsten Werner, Bàrbara Laviña, Luka Kulic, Roger M. Nitsch, Christian Tackenberg, Martin E. Schwab

**Affiliations:** ^1^Institute for Regenerative Medicine, University of Zurich, Zurich, Switzerland; ^2^Department of Health Sciences and Technology, ETH Zürich, Zurich, Switzerland; ^3^Neuroscience Center Zurich, University of Zurich and ETH Zürich, Zurich, Switzerland; ^4^Leibniz Institute for Polymer Research, Dresden, Germany; ^5^Department of Immunology, Genetics and Pathology, Uppsala University, Uppsala, Sweden

**Keywords:** quantification, image processing, angiogenesis, blood vessels, stroke, development, central nervous system (CNS)

## Abstract

The distinct organization of the brain’s vasculature ensures the adequate delivery of oxygen and nutrients during development and adulthood. Acute and chronic pathological changes of the vascular system have been implicated in many neurological disorders including stroke and dementia. Here, we describe a fast, automated method that allows the highly reproducible, quantitative assessment of distinct vascular parameters and their changes based on the open source software Fiji (ImageJ). In particular, we developed a practical guide to reliably measure aspects of growth, repair and maturation of the brain’s vasculature during development and neurovascular disease in mice and humans. The script can be used to assess the effects of different external factors including pharmacological treatments or disease states. Moreover, the procedure is expandable to blood vessels of other organs and vascular *in vitro* models.

## Introduction

Structural and functional integrity of blood vessels are essential for normal brain function. A complex vascular network supplies the brain with oxygen and nutrients, removes metabolic waste products, conveys hormonal signaling and allows rapid distribution of immune cells (Rust et al., 2018, 2019c; Sweeney et al., 2018). As opposed to the periphery, brain vasculature also forms a highly selective border, the BBB (Zhao et al., 2015; Sweeney et al., 2019b). The BBB consists of a continuous brain microvascular endothelium, its underlying basement membrane, pericytes, astrocytes and microglia. It maintains the homeostatic environment within the brain, which is required for proper functioning of neuronal circuits (Sweeney et al., 2016; Iadecola, 2017; Brown et al., 2019).

Since the brain is highly vulnerable to compromises of blood supply, brain vasculature has long been an object of scientific interest due to its central role in major neurovascular diseases including strokes and dementia. Correct interpretation of structural changes in the vasculature is therefore fundamental to understand its underlying (patho-)physiologies (Potente and Carmeliet, 2017; Sweeney et al., 2018). Advances in high resolution laser microscopy and *in vivo* imaging techniques reinforced the use of image-based analysis in vascular research for both animal experiments and human pathologies (Ertürk et al., 2014; Zhang et al., 2018). Despite improvements in imaging techniques, interpretation of these data can be challenging, time-consuming and laborious due to the 3D nature and high complexity of the brain vasculature. A consequence are poor levels of quantification and high variation between analysis methods and investigators.

Computational methods have facilitated the quantification of vascular networks. However, most of them currently rely on commercial software (e.g., Matlab, Imaris, Vesselucida) that can be expensive and require considerable expertise (Walter et al., 2010; Reyes-Aldasoro et al., 2011; Craver et al., 2016; Montoya-Zegarra et al., 2019). Alternatives are open-source packages that also provide excellent extensions tailored for specific applications to trace and analyze the features of the vasculature. Nonetheless, many extensions are not well maintained and might not be compatible with one another.

Here, we provide a straightforward practical guideline how to process vascular images, identify biologically relevant vascular parameters and automatize these processes with a script based on the open source Fiji (ImageJ) software. The script implements pre-processing steps to binarize the raw image and visualize it with a heatmap. Quantitative assessment provides information about the vascular area fraction, vascular segment length, number of branch points and the distance and distribution between single vessels. Moreover, we have developed a pipeline how to measure pericyte coverage of the vasculature that has been shown to be a critical factor for vascular integrity and physiological function. All features have been tested for compatibility across different ImageJ versions. The script is applicable to vascular development and vascular pathologies in mice and humans but has also been validated on retinal vascular development *in vitro* models.

## Results

### Quantification of Key Anatomical Features of the Developing Brain Vasculature

**FIGURE 1:**
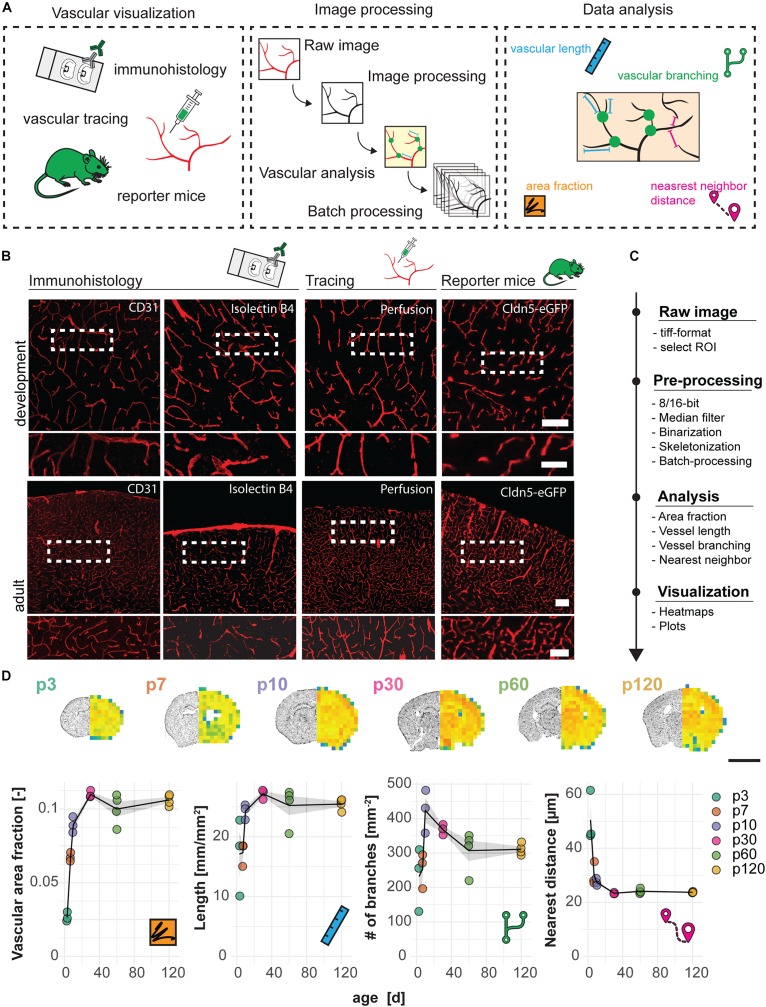
Visualization and quantification of cortical vasculature in the developing mouse brain. **(A)** Experimental design. **(B)** Visualization of the vasculature with immune- or lectin-histofluorescence, transcardial perfusion with Lectin-Dylight594 or the use of Cldn5-GFP reporter mice at the age of p10 (development) and 3 months (adult). Scale bar 50 μm. **(C)** Step-by-step analysis pipeline for vascular quantification. **(D)** Heatmap and quantification of vascular area fraction, length, branching and nearest distance between blood vessels in cortical brain sections. Scale bar: 2 mm. Data are represented as means ± SD. Each dot represents one animal.

Mouse brain vasculature can be visualized by different procedures such as the use of specific ligands or immunohistological markers (e.g., lectins or antibodies against CD31), intravascular perfusion with fluorophore-coupled substances (e.g., Lectin Dylight594) or the use of genetic mouse models that express a reporter under the control of a vascular-specific gene (e.g., Cldn5-GFP). Here, we show that these methods result in the specific visualization of the brain vasculature, and therefore are suitable to perform quantitative analyses with our automated image processing and data analysis pipeline (Figures 1A,B). All methods led to visualization of the entire vascular system including the smallest vessels and newly formed capillaries, including endothelial tip cells (Supplementary Figure S1).

As an example, images of cortical regions from Cldn5-GFP mice at the age of postnatal day p3, p7, p30, p60, and p120 were processed and automatically analyzed for the vascular area fraction, vessel segment length, vascular branching and the distance between single vessels (according to Script in Supplementary Figure S2) with the developed Fiji (ImageJ) protocol (Figures 1C,D). The analysis revealed a steady increase of vascular area fraction (p3: 0.027 ± 0.003; p120: 0.106 ± 0.004, *p* < 0.001), vascular length in mm per mm2 (p3: 17.12 ± 6.45; p120: 25.47 ± 0.95, *p* = 0.028), number of vascular branches per mm2 (p3: 232.09 ± 92.16; p120: 310.74 ± 16.29, *p* = 0.449); and a decrease in distance between the vessels in μm (p3: 50.49 ± 9.50; p120: 23.72 ± 0.23, *p* < 0.001). Interestingly, we also observed a continuous reduction in the number of branch points per mm^2^ starting from p10, indicating the decrease in anastomoses that is known to occur during normal development and maturation of the vasculature (Wang et al., 1992) (p10: 423.43 ± 62.37; p120: 310.74 ± 16.29, *p* = 0.137) (Figure 1D).

In addition to cerebrovascular development, we confirmed the applicability of our automated analysis pipeline for blood vessel development in the retina from postnatal day 3 to 120 (Supplementary Figure S3). Moreover, we also validated its application for *in vitro* systems of 3D vascular networks derived from human umbilical vein endothelial cells (HUVECs) grown in a matrix with and without inhibitory factors for vascular growth (Supplementary Figure S4).

### Disruption of Cerebral Vascular Network and Pericyte Coverage Following Ischemic Stroke

Ischemic strokes (the most frequent kind of vascular failure in the brain) lead to a partially blocked and disrupted vascular network. Often, a central stroke core with severely compromised blood flow develops, surrounded by a rim of partially ischemic tissue (ischemic border zone) with impaired function but in part preserved cellular viability (Sweeney et al., 2018).

**FIGURE 2:**
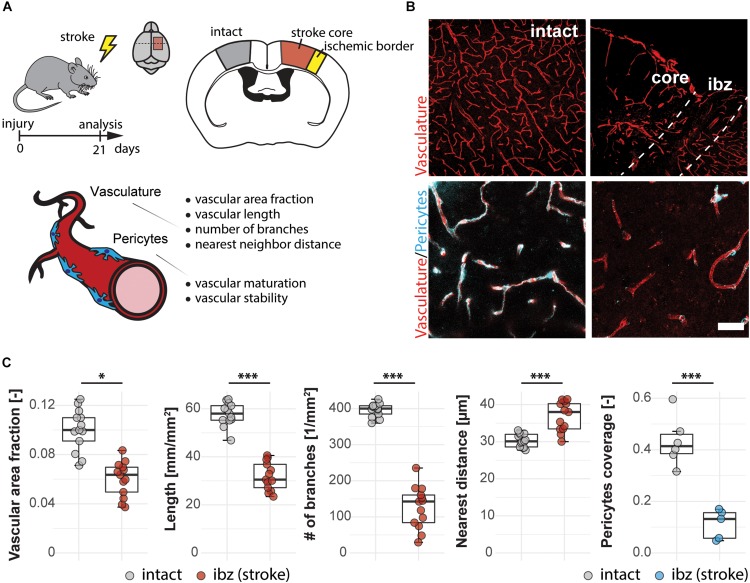
Vascular changes in the peri-infarct ischemic border zone around the stroke core in the mouse cortex. **(A)** Experimental design. **(B)** Representative images of intact cortex vasculature (left panels) and endothelial network (CD31+; red) and pericytes (CD13+; blue) in the ischemic border zone. Scale bar: 100 μm (overview), 20 μm (close-up). **(C)** Quantitative assessment of vascular parameters (area fraction, length, branching, distance) and pericyte coverage of the vasculature. Data are represented as boxplots (median, two hinges and two whiskers); the upper and lower hinges correspond to the first and third quantiles. The whiskers extend until 1.5 × IQR from the hinges; data beyond the end of the whiskers are defined as outlying points. Each dot represents one animal, and significance of mean differences between the groups was assessed using two-tailed unpaired one-sample *t*-test. Asterisks indicate significance: ^∗^*P* < 0.05, ^∗∗∗^*P* < 0.001. IQR, interquartile range.

Here, we use the photothrombotic stroke model in the sensorimotor cortex of mice to analyze the vasculature in the ischemic border zone 3 weeks following stroke (Figures 2A,B). We detected changes in all vascular parameters between the intact and the peri-infarct cortex (Figure 2C). There was a decrease in vascular area fraction (−39%; intact: 0.100 ± 0.017; injury: 0.060 ± 0.014, *p* < 0.001), vessel segment length in mm/mm2 (−44%; intact: 57.74 ± 4.83, injury: 31.89 ± 5.83, *p* < 0.001), number of branches per mm2 (−68%; intact: 396.31 ± 18.81, injury: 126.57 ± 58.30, *p* < 0.001). Moreover, the distance between the vessels in μm increased in the peri-infarct region (+21.4%; intact: 30.34 ± 1.76, injury: 36.85 ± 3.96, *p* < 0.001).

Pericyte coverage is an important parameter for physiological function and maturity of cerebral blood vessels. As perivascular multi-potent cells and an important component of the BBB (Sweeney et al., 2018, 2019b), pericytes have been shown to play important roles during vascular growth and maturation, immune functions and BBB maintenance (Zlokovic, 2010; Gautam and Yao, 2018). Therefore, we included the analysis of the vascular pericyte coverage in our script. At 3 weeks after the stroke, the peri-infarct regions around the stroke core still showed a 74% lower pericyte coverage (0.112 ± 0.056) compared to the intact hemisphere (0.432 ± 0.096, *p* < 0.001).

### Vascular Pericyte Coverage Is Reduced in Alzheimer’s Patients in the Medial Frontal Cortex

Vascular changes and BBB disruption are also major contributors of Alzheimer’s disease pathophysiology and therefore widely investigated with post-mortem studies (Nelson et al., 2016). Within these changes, decreased pericyte coverage of vessels was reported by various groups both in transgenic animal models and in human brain samples (Visser et al., 1990; Halliday et al., 2016; Ma et al., 2018; Miners et al., 2018).

**FIGURE 3:**
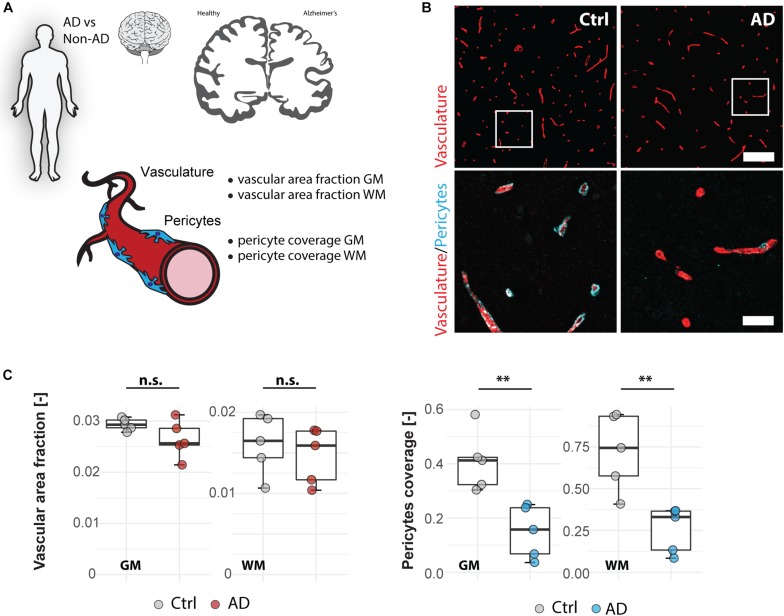
Vascular changes in human Alzheimer’s disease brains. **(A)** Experimental design. **(B)** Representative images of Alzheimer’s brain sections. Scale bar 100 μm (overview), 20 μm (close-up). **(C)** Quantitative assessment of brain vasculature and pericyte coverage in cortical gray and white matter sections of AD patients and healthy controls. Data are represented as boxplots (median, two hinges and two whiskers); the upper and lower hinges correspond to the first and third quantiles. The whiskers extend until 1.5 × IQR from the hinges; data beyond the end of the whiskers are defined as outlying points. Each dot represents one subject and significance of mean differences between the groups was assessed using a two two-tailed unpaired one-sample *t*-test. Asterisks indicate significance: ^∗∗^*P* < 0.01. GM, gray matter; WM, white matter; AD, Alzheimer’s disease; Ctrl, control; n.s., not significant; IQR: interquartile range.

To test our automated image processing pipeline in a human post-mortem vascular analysis, we applied the script for quantification of the vascular area fraction and pericyte coverage in gray and white matter tissue samples of the medial frontal cortex (Figures 3A,B) from five Alzheimer patients and five age-matched controls (Supplementary Table S1). In line with the earlier reports (Nyúl-Tóth et al., 2016; Kubíková et al., 2018; Hase et al., 2019), our analysis showed a higher vascular area fraction in GM compared to white matter (GM: 0.028 ± 0.003; WM: 0.015 ± 0.003, *p* < 0.001) (Figure 3C) whereas pericyte coverage was not significantly different between gray and white matter (GM: 0.279 ± 0.168, WM: 0.489 ± 0.304, *p* = 0.078) (Figure 3C). Quantification of vascular area fraction didn’t reveal any significant changes between non-demented controls and AD patients in neither cortical GM (Ctrl 0.029 ± 0.001, AD: 0.026 ± 0.003, *p* = 0.130), nor white matter regions (Ctrl: 0.016 ± 0.004, AD: 0.015 ± 0.003, *p* = 0.554) (Figure 3C). On the other hand, pericyte coverage showed a significant reduction of PDGFR-β immunoreactivity in AD patients compared to controls both in the GM: −63% (Ctrl: 0.408 ± 0.110, AD: 0.149 ± 0.097, *p* = 0.004) and in the white matter −64% (Ctrl: 0.721 ± 0.231, AD: 0.256 ± 0.137, *p* = 0.004). Thus, our automated analysis pipeline confirmed the reduction in pericyte coverage in AD patients both in white and GM of the medial frontal cortex, but the overall vascular density seemed not be affected in our patient sample.

## Discussion

We describe a practical toolbox based on open-source software for the analysis of complex blood vessel networks during development and following cerebrovascular diseases. The pipeline allows to quantitatively assess the important parameters vascular area fraction, vessel segment length, vascular branching, nearest distance between single vessels and pericyte coverage in a fast and reliable way in developing, adult and diseased brains of mice and humans. Moreover, we validated the versatile applicability of the toolbox for mouse retinal tissue and vascular *in vitro* models.

Reliable quantification of the vasculature and its supporting cells is fundamental to detect pathological changes in several major diseases. Consequently, many research institutes and pharmaceutical companies are assessing drugs and treatments to either predict early changes in the vasculature or to find an effective treatment. In the field of cancer, tumor growth is strongly promoted by the formation of new vessels – and treatment strategies are developed to inhibit vascular growth (Rust et al., 2018; Alcala et al., 2019; Jiang et al., 2019). In diabetic retinopathy (DR), a major vascular complication of diabetes mellitus, the dysfunction is characterized by the formation of new vessels inside the retina. New mechanisms and targets are currently discovered to alleviate the vascular pathology in DR (Zhu et al., 2019a). Moreover, alterations in the vasculature and vascular supporting cells have been proven to be a hallmark of hypertension, atherosclerosis and advanced chronic liver disease (Li et al., 2019; Maeso-Díaz et al., 2019; Miao et al., 2019). In the CNS, regulation of vascular remodeling and repair has been shown influence pathological progression of e.g., stroke, cerebral small vessel disease, Parkinson’s, and Alzheimer’s (Wei et al., 2016; Zou et al., 2017; Shen et al., 2019; Sweeney et al., 2019a; Zhu et al., 2019b). Consequently, the development of tools to reliably describe early changes in the vascular anatomy are relevant for a large variety of research.

Comparison of different visualization methods for brain blood vessels including immune- and lectin-histochemistry, vascular tracer perfusion and genetic reporter mice revealed similar patterns of the brain blood vessels. Nonetheless, since perfusion methods only label vasculature that is supplied with blood, newly formed blood vessels with their tip cells and filopodia remain undetected. by intravascular tracer perfusion. Methods may be chosen dependent on the experimental questions and set up.

Several methods were proposed for the quantitative assessment of vascular networks (Seaman et al., 2011; Zudaire et al., 2011; Zhang et al., 2018; Montoya-Zegarra et al., 2019). However, most rely on commercial, often expensive software, may require considerable expertise or are specialized for specific vascular networks, e.g., the retina (Walter et al., 2010; Reyes-Aldasoro et al., 2011; Craver et al., 2016; Montoya-Zegarra et al., 2019).

A main advantage of the present toolbox is that it is free of charge, easy and straightforward in practice and has a broad application. It does not require any technical knowledge in coding to adapt the script. Moreover, it has been tested and validated across different operating systems and ImageJ versions. It only requires the initial installation of two plugins for the measurement of vessel segment length (Measure Skeleton Length) and distance calculations (Nearest Neighbor Distances Calculation with ImageJ) before starting. The toolbox covers the entire process of image processing, visualization and analysis of relevant biological parameters. We decided to include a quantitative assessment of vascular area fraction for a general overview of vascular density. In addition to vessel density, length and branching are relevant for an understanding of exchange of oxygen and nutrients between the capillary network and the brain tissue (Kopylova et al., 2017). The distance and its variability between blood vessels additionally allows the detection of local hypoxic regions that may not entirely be represented by the overall mean vascular density. Previous versions of the script have been used to assess post-stroke angiogenesis and retinal vascular growth (Rust et al., 2019a, b, d). Interestingly, we observe very high levels of vascular density in the adult mouse retina (higher than in the mouse brain), which has been previously observed (Chintala et al., 2015; Zhang et al., 2018; Rust et al., 2019a, d).

The measured pericyte coverage of blood vessels in our samples (both human and mice) ranged between 40–60% and has been previously reported to be around 80% (Nikolakopoulou et al., 2017; Montagne et al., 2018). These discrepancies are likely to occur due to the use of different antibodies and/or different epitopes (CD13, PDGFRβ for pericytes or CD31, lectin, Laminin, Collagen IV for vasculature). Moreover, further studies are needed to evaluate differences between animal Alzheimer’s disease (AD) models and a large cohort of human AD samples. The presented study was designed to evaluate and validate the automated script for vascular analysis. Accordingly, we had only 5 human AD subjects and 5 non-demented controls. All AD subjects were female, APOE E4 carriers and had a Braak Score of 5–6. Larger cohorts are required to reliably interpret the observed vascular and pericyte phenotypes between AD and control cohorts.

The performed immunostainings resulted in a uniform visualization of all blood vessels; however, we did not intent to distinguish between different types of blood vessels (such as arteries, arterioles, capillaries, venules, and veins). Several antigens were previously reported to be expressed specifically in either veins or arteries (Kretschmer et al., 2013) and might be also used for analysis. The sensitivity and reliability of the script strongly depends on the quality of vascular visualization and imaging. To our experiences we observe the best signal to noise ratio by using transgenic models or performing transcranial perfusion with fluorescent dyes. We have observed that especially old human tissue might show heterogeneity between brains and therefore all automated steps should be manually controlled.

A limitation of the script is that it does not provide information about the vascular integrity. Many neurological diseases including stroke and Alzheimer’s disease have damage of the BBB and extravasation of blood-derived neurotoxic molecules as a hallmark. There are many approaches to study BBB integrity in mice and human including the use of vascular fluorescent tracers or immunostaining of extravasated endogenous molecules e.g., fibrinogen. Especially in human, BBB integrity can also be assessed using magnetic resonance imaging (MRI) and NIRS. Automated quantification of BBB integrity has been previously described (Michalski et al., 2010; Tang et al., 2010; Saunders et al., 2015).

Vascular damage is an important hallmark of many neurological diseases, but, the underlying changes in vascular anatomy and physiology may differ. Studies should ideally combine structural end-point measures with physiological data from the different brain imaging techniques including functional MRI (fMRI) and PET (Mier and Mier, 2015).

## Conclusion

We established and validated a straightforward and versatile toolbox based on the open-source software ImageJ to reliably describe brain vasculature in tissue sections. It is applicable to the developmental, adult and diseases vasculature and has been also tested outside the CNS. The protocol will facilitate the detection of pathological changes or results of genetic or pharmacological manipulations of the vascular anatomy.

## Materials and Methods

### Animals

We used wildtype and Claudin-eGFP (Cldn5-GFP) C57BL/6^13^ mice. Cldn5-GFP were generated using bacterial artificial chromosome (BAC) technology. Animals were maintained at the Brain Research Institute and at the Laboratory Animal Services Center in Zürich on a 12 h light/dark cycle with food and water provided *ad libitum*. Mice were housed in standard Type II/III cages at least in pairs. Animals used were 7 days to 3-month-old males and females. All experiments were conducted in accordance with the applicable national regulations and approved by the Cantonal Veterinary Department of Zürich.

### Human Brain Tissue

Paraffin-embedded middle-frontal cortex tissue blocks were provided by the Netherlands Brain Bank (NBB), the Netherlands Institute for Neuroscience, Amsterdam, Netherlands. Post-mortem samples were collected from donors with a written informed consent for brain autopsy and the use of the material for research purposes was obtained by the NBB. Braak stage (Braak and Braak, 1995), CERAD (Consortium to Establish a Registry for Alzheimer’s Disease) score for amyloid load (Hyman et al., 2012), APOE genotype and clinical diagnosis for each subject were determined and provided by NBB. Demographics of all subjects were listed in Supplementary Table S1.

### Tissue Processing for Mouse Brain Sections

Animals were deeply anesthetized by intraperitoneal injection of pentobarbital (150 mg/kg bodyweight) and subsequently transcardially perfused with isotonic Ringer solution (5 ml/l Heparin, B. Braun) and 4% Paraformaldehyde (PFA, in 0.2 M phosphate buffer, pH 7.4). Brains were removed from the skulls and post-fixed for 4 h at 4°C. For cryoprotection, brains were transferred to 30% sucrose and kept overnight at 4°C. Coronal sections with a thickness of 40 μm were cut using a sliding microtome (Microm HM430, Leica). Sections were collected and stored as free-floating sections in cryoprotectant solution at −20°C until further processing. For immunohistochemical staining brain sections were washed with 0.1 M phosphate buffer (PB) and then incubated with a blocking and permeabilization solution (TNB, 0.1% TBST, 3% normal goat serum) for 30 min at RT shaking. Sections were incubated with primary antibody (rat-CD31, BD Biosciences, 1:100) overnight at 4°C. The next day sections were washed and incubated with corresponding secondary antibodies for 2 h at RT. All antibodies were diluted in blocking and permeabilization solution (TNB, 0.1% TBST, 3% normal goat serum). Nuclei were counterstained with DAPI (1:2000 in 0.1 M PB). Sections were mounted in 0.1 M PB on Superfrost PlusTM microscope slides (Thermo Fisher) and coverslipped using Mowiol.

### Tissue Processing for Human Brain Sections

Five μm thick sections of paraffin-embedded human middle frontal cortex blocks were prepared with Leica RM2135 rotary microtome and mounted on slides. Sections were placed in xylol, 100% ethanol, 95% ethanol, 70% ethanol and water in respective order and kept in each solvent for 3 min. For antigen retrieval, sections were boiled in 0.1M citrate buffer. After washing with phosphate-buffered saline (PBS), sections were blocked with 10% horse serum in PBS with 0.2% Triton X-100 (PBS-T) for 2 h at room temperature. After blocking, primary antibody cocktail (1:500 dilution of biotinylated-lectin (B-1065, Vector Laboratories) and 1:50 dilution of goat anti-PDGFR-β antibody (AF385, R&D systems) in 5% horse serum in PBS-T) was applied and incubated overnight at 4°C. Next day, sections were incubated for 2 h at room temperature with secondary antibodies [1:200 dilution in 5% horse serum in PBS-T, streptavidin conjugated to Alexa488 and donkey anti-goat conjugated to Cy3 (Jackson Immunoresearch)] and for 5 min with 0.2% Sudan Black (Sigma Aldrich) in 70% ethanol. Coverslips were mounted with Histomount (National Diagnostics) mounting medium. No white matter lesions were observed in AD samples.

### Transcardial Perfusion With Vascular Tracers

After deep anesthesia and shortly before perfusion, 50 μl of 1 mg/ml Lycopersicon Esculentum lectin conjugated to DyLight594 (DL-1177, VactorLabs) were transcardially injected. After 1 min animals were transcardially perfused with isotonic Ringer solution (5 ml/l Heparin, B. Braun) and 4% Paraformaldehyde (PFA, in 0.2 M phosphate buffer, pH 7.4.

### Photothrombotic Stroke

Animals were anesthetized with 2% isoflurane followed by an intraperitoneal injection of fentanyl (20 ug/kg), midazolam (1 mg/kg), medetomidine (200 ug/kg). A photo-thrombotic stroke to unilaterally lesion the sensorimotor cortex was introduced on the right hemisphere. Animal was fixed in a stereotactic frame, the skull exposed through a midline incision, cleared of connective tissue and dried. A cold light source (Olympus KL 1500LCS, 150W, 3000 K) was positioned over an opaque template with an opening for the light source (5 × 3 mm) 2.5 mm to −2.5 mm anterior and 0 mm to 3 mm lateral to Bregma. Rose Bengal (13 mg/kg body weight, 10 mg/ml Rose Bengal in 0.9% NaCl solution) was intraperitoneally injected and after 5 min, the brain was illuminated through the intact skull for 8 min. For postoperative care, all animals received analgesics (Dafalgan Sirup, Braun, *per os* in the drinking water) and antibiotics (Baytril, 5 mg/kg body weight, Bayer, s.c.) once a day for at least 3 days after surgery. Sham operated animals underwent the entire surgical process but did not received Rose Bengal. Vascular analysis was performed 3 weeks following stroke injury; however, it has also been validated to other time points (day 1, 3, 7, 28, data not shown).

### Microscopy and Vascular and Pericyte Quantification

Imaging of brain sections was performed with Leica SP8 laser scanning confocal microscope equipped with 10x, 20x, 40x objectives. Images were processed using Fiji (ImageJ) and Adobe Illustrator CC. First, regions of interest were chosen for the respective experiments. The regions were defined as stroke core (no surviving neurons), ischemic border zone (ibz; a region of 300 um distal to the stroke core with hypovascularization) and intact (cortical contralesional site) as previously described (Rust et al., 2019b, d). Images were pre-processed and binarized according to Figure 1. To assess vascular growth and repair an ImageJ (Fiji) script was established to automatically calculate (1) area fraction of blood vessels, (2) vessel segment length (3) vascular branching (4) distance between blood vessels. Maturation of blood vessels was assessed by the ratio of pericyte coverage to total vascular area fraction. To assess the information images are duplicated, processed (to remove noise) and binarized. Area fraction calculation measures the percentage of pixels with non-zero pixels. Vessel segment length and vascular branching is assessed by skeletonizing the binary image. This allows to tag all pixels in a skeleton image and then count all its branches and measures their average and maximum length. The distance between single vessels is calculated by the nearest neighbor distance that displays the distance between closest individual single non-zero pixels. Quantification of the pericyte coverage is measured by dividing the pericyte area fraction signal from the previously acquired vascular area fraction signal. The relative ratio provides the pericyte coverage of the blood vessels. Heatmap analysis is performed by selecting the number of squared per row/column. For each square the area fraction is calculated (as described above) and the values are visualized with a heatmap. The size of squared is adjustable and should be adapted to the used magnification and sample. The corresponding script can be found in Supplementary Data Sheet S1.

### Hydrogel Formation

Biohybrid GAG-PEG hydrogels for *in vitro* HUVEC morphogenesis studies were prepared as described previously with slight modifications. In brief, a degradable starPEG-MMP conjugate (MW 16,489) was dissolved in HUVEC culture medium at a final concentration of 0.75 mM and a heparin-maleimide conjugate (MW 15,000) was dissolved in the medium and supplemented with the adhesive peptide CWGGRGDSP (cRGD, MW 990) to reach a final concentration of 1 mM and 2 mM, respectively. Thereafter, the heparin-RGD mixture was (non-reactively) functionalized with VEGF165 (PeproTech, United States), SDF1 (Miltenyi Biotec, Germany) and FGF2 (Miltenyi Biotec, Germany) each at a final concentration of 5 μg/ml. The myelin was added in concentrations of rat Myelin at 10 μg/ml) before adding a HUVEC suspension to generate a cell-heparin conjugate mixture. For the formation of hydrogels, the cell-heparin conjugate mixture was mixed with the starPEG conjugate solution in a 1:1 volume ratio to form 15 μl hydrogel droplets which were casted onto hydrophobic 12 well chamber slides (Ibidi, Germany). Following the *in situ* crosslinking, the gels were immediately immersed in cell culture medium and 70% of the medium was exchanged with fresh medium every other day. Three to five replicates per condition were produced for all seven conditions investigated. On day 3 the samples were fixed with 4% paraformaldehyde for 15 min at RT and immunostained thereafter.

### Immunocytochemistry for *in vitro* Experiments

After fixation and washing with PBS the samples were permeabilized using 0.1% Triton X-100 for 10 min. The samples were blocked with 2% BSA and 2% goat serum in PBS for 3 h and incubated over night with the primary antibodies mouse anti-CD31 (BD 555444; 1:100) at 4°C. After 3 washes with PBS/0.1% BSA, the secondary antibody Alexa Flour 488 goat anti-mouse (Life Technologies 1:200) was added and incubated over night at 4°C. Samples were washed and incubated with Hoechst 33342 (Life Technologies; 1:200) over night at 4°C. Fluorescent images were captured using a Dragonfly Spinning Disk confocal microscope (Andor). At least three images were taken at different positions within each hydrogel. Fields and samples for imaging and quantification were chosen randomly for all experiments.

### Statistical Analysis

Statistical analysis was performed using RStudio. Sample sizes were designed with adequate power according to our previous studies and to the literature. All data were tested for normal distribution by using the Shapiro–Wilk test. Normally distributed data was tested for differences with a two-tailed unpaired one-sample *t*-test to compare differences between two groups (vascular repair in stroke mice, retinal development, *in vitro* model, Alzheimer’s patients). Multiple comparisons were assessed using ANOVA followed by Tukey’s HSD *post hoc* test (time course of developmental brain vasculature). Data are expressed as mean ± SD and statistical significance was defined as ^∗^*p* < 0.05, ^∗∗^*p* < 0.01, and ^∗∗∗^*p* < 0.001.

## Data Availability Statement

The datasets generated for this study are available on request to the corresponding author.

## Ethics Statement

The animal study was reviewed and approved by the Cantonal Veterinary Department of Zurich. The studies involving human participants were reviewed and approved by the Netherlands Brain Bank. The patients/participants provided their written informed consent to participate in this study.

## Author Contributions

RR, TK, and MS designed the study, prepared the figures, and wrote the manuscript. RR, TK, LG, BD, YL, and AM carried out the experiments. TK, BL, LK, RN, CW, and CT provided tissue and/or the Cldn5-eGFP mouse model. TK, LG, MS, and CT proof-read and revised the manuscript. All authors read and approved the final manuscript.

## Conflict of Interest

The authors declare that the research was conducted in the absence of any commercial or financial relationships that could be construed as a potential conflict of interest.

## Abbreviations

BBB: blood brain barrier
Cldn5: claudin-5
CNS: central nervous system
fMRI: functional magnetic resonance imaging
GFP: green fluorescent protein
GM: gray matter
NIRS: near-infrared spectroscopy
PET: positron emission tomography
ROI: region of interest
WM: white matter.
