# Supplementary material for: A Practical Guide to the Automated Analysis of Vascular Growth, Maturation and Injury in the Brain
Source: Front Neurosci. 2020 Mar 20;14:244. doi: 10.3389/fnins.2020.00244 (PMC7099171; doi:10.3389/fnins.2020.00244)
Supplement: Supplementary file 1 [file Data_Sheet_1.zip › RR_Suppl_Script_Vascular_Analysis/IMAGE_J_SUPPL_DATA/Supplementary data sheet 1.docx]

**Supplementary data sheet 1: Analysis toolbox for vascular quantification in ImageJ (Fiji).** The toolbox consists of two files for quantification of the vasculature (Quantification.ijm), a script for heatmap generation (Heatmap.ijm), an example image for quantification (representative image.tif) and a lut-file for heatmap generatio n(hm_stroke.lut)
