# Supplementary material for: A Practical Guide to the Automated Analysis of Vascular Growth, Maturation and Injury in the Brain
Source: Front Neurosci. 2020 Mar 20;14:244. doi: 10.3389/fnins.2020.00244 (PMC7099171; doi:10.3389/fnins.2020.00244)
Supplement: Supplementary file 2 [file Table_1.docx]

Supplementary Table 1: Demographics of human post-mortem brain samples

| **Subject no** | **Braak Stage** | **CERAD^a^ Score** | **APOE^b^ Genotype** | **Age at Death** | **Gender** | **PMD^d^** | **Clinical Diagnosis** |
| --- | --- | --- | --- | --- | --- | --- | --- |
| Subject 1 | 0 | A | N/A^c^ | 90 | Male | 07:40 | Non-demented Control |
| Subject 2 | 0 | O | E3/E3 | 80 | Male | 07:15 | Non-demented Control |
| Subject 3 | 0 | B | E3/E3 | 88 | Female | 06:15 | Non-demented Control |
| Subject 4 | 1 | B | E3/E3 | 69 | Female | 15:30 | Non-demented Control |
| Subject 5 | 1 | A | E3/E3 | 87 | Male | 10:20 | Non-demented Control |
| Subject 6 | 5 | C | E3/E4 | 69 | Female | 05:45 | Alzheimer’s Disease |
| Subject 7 | 6 | C | E3/E4 | 89 | Female | 04:30 | Alzheimer’s Disease |
| Subject 8 | 6 | C | E4/E4 | 86 | Female | 05:00 | Alzheimer’s Disease |
| Subject 9 | 6 | C | E3/E4 | 94 | Female | 05:40 | Alzheimer’s Disease |
| Subject 10 | 6 | C | E3/E3 | 91 | Female | 03:40 | Alzheimer’s Disease |

^a^CERAD: Consortium to Establish a Registry for Alzheimer’s Disease

^b^APOE: Apolipoprotein E

^c^N/A: Not available

^d^PMD: Post-mortem delay
